# Isavuconazole in the treatment of acute invasive fungal sinusitis: two case reports and literature review

**DOI:** 10.3389/fmed.2025.1700685

**Published:** 2026-01-12

**Authors:** Kun Zhao, Yuwan Song, Huhuifen He, Fengxin Yang, He Zhao, Yan Wang, Li Wang, Yan Wang, Yan Sun

**Affiliations:** 1 The Second Medical College, Binzhou Medical University, Yantai, Shandong, China; 2 Department of Otorhinolaryngology, Head and Neck Surgery, Yantai Yuhuangding Hospital, Qingdao University, Qingdao, China; 3 Shandong Provincial Key Laboratory of Neuroimmune Interaction and Regulation, Yantai, China; 4 Shandong Provincial Clinical Research Center for Otorhinolaryngologic Diseases, Yantai, China; 5 Yantai Key Laboratory of Otorhinolaryngologic Diseases, Yantai, China; 6 Department of Otorhinolaryngology, Head and Neck Surgery, Charité - Universitätsmedizin Berlin, Corporate Member of Freie Universität Berlin and Humboldt Universität zu Berlin, Berlin, Germany

**Keywords:** Mucor, Aspergillus, fungus, isavuconazole, AIFRS

## Abstract

**Objective:**

Through the analysis of two successfully treated cases of acute invasive fungal rhino-sinusitis (AIFRS) caused by Mucor in our hospital, to enhance the understanding of this disease and explore improved diagnostic and treatment strategies. This study also conducted a comprehensive literature review of AIFRS. This study also provides a comprehensive literature review of Infections of the mucor and aspergillus genera.

**Methods:**

The clinical data of two patients with AIFRS admitted to our hospital from 2022 to 2025 were retrospectively analyzed, focusing on risk factors, clinical manifestations, treatment plans, and outcomes. Additionally, we reviewed and analyzed cases of Infections of the Mucor and Aspergillus genera published in PubMed over the past 15 years (2011–2025).

**Results:**

A total of 387 cases were included in the analyzed studies. The patient cohort was systematically categorized into two main groups: those with Mucor infection and those with Aspergillus infection. Within the Mucor infection group, four subgroups were further identified. The average age of patients infected with Mucor was 41 ± 31.11 years, while that of patients infected with Aspergillus was 32.5 ± 29.99 years. Since the sample size in some subgroups was less than 5 cases, we chose Fisher’s exact test to calculate whether there were significant differences, the efficacy rate of isavuconazole monotherapy (89.47%) was significantly higher than that of amphotericin B monotherapy (59.68%), *p* < 0.05. Similarly, the efficacy rate of isavuconazole combination therapy (86.67%) was significantly superior to that of amphotericin B monotherapy (59.68%), *p* < 0.05. Amphotericin B combination therapy demonstrated a marginally better efficacy rate (76.92%) compared to amphotericin B monotherapy (59.68%), *p* < 0.05.

**Conclusion:**

In the described cases, ours patients received isavuconazole pre- and post-surgery, achieving favorable prognostic outcomes. Literature comparisons further validate these findings, demonstrating that the efficacy of the new triazole drug isavuconazole in treating invasive aspergillosis and mucormycosis significantly surpasses that of amphotericin B, with efficacy comparable to voriconazole (*p* < 0.05). These results provide valuable guidance for clinical medication decisions.

## 1 Introduction

Acute invasive fungal rhino-sinusitis (AIFRS) is a rare but aggressive opportunistic infection originating in the paranasal sinuses. It can invade the sinus mucosa, bone walls, and adjacent structures such as the eyes and skull base, leading to intracranial and extracranial complications (1–3). The disease typically progresses within 4 weeks and primarily affects immunocompromised patients, such as those with leukemia, diabetes, acquired immune deficiency syndrome (AIDS), or those undergoing anti-tumor treatments, organ transplantation, radiotherapy, or chemotherapy (4, 5). Clinical manifestations are non-specific and include facial swelling, nasal congestion, rhinorrhea, fever, headache, and facial pain (5–7). Affected patients demonstrate some degree of compromised immune function and tend to be critically ill (8, 9). Common causative fungi include Mucor, Rhizopus, and Aspergillus, while rare species include Alternaria, Candida, and Fusarium. Mucor and Aspergillus can invade blood vessels, with Mucor being more invasive, particularly into nerve vessels and the orbit (10–12). Aspergillus tends to predominate in neutropenic patients, whereas Mucor is more common in poorly controlled diabetic patients, especially those with diabetic ketoacidosis or receiving Deferoxamine therapy (9, 13, 14). Despite advancements in antifungal drugs and surgical techniques, the mortality rate remains high, ranging from 33 to 80% (15, 16). We report two surviving cases of AIFERS treated with isavuconazole and review the literature.

## 2 Materials and methods

We searched PubMED^1^ in January 2025 for articles describing cases of severe infections caused by the mucor and aspergillus genera in populations published since 2011. We used keywords such as “mucor,” “aspergillus,” “fungus,” “isavuconazole,” and relevant synonyms to broaden our results. Our main inclusion criteria were (1) a focus on all populations, (2) Prove that it is these fungal strains that lead to all kinds of serious infections, (3) the severity and clinical significance of the infection, and (4) the clinical focus of the article, with information on manifestations and outcome. We excluded articles that (1) information on cases could not be extracted; (2) did not clearly report evidence of fungal strains; (3) non-eligible study type or unrelated to research question. To refine our search, we applied filters, selecting our population of interest and articles published in English. No automation tools were used. Finally, we utilized Microsoft Excel to organize the collected data into tables and generate corresponding charts, followed by statistical analysis conducted with SPSS software. Given that certain subgroups included in the final statistical analysis consisted of fewer than five cases, Fisher’s exact test was employed to minimize potential errors arising from the statistical methodology. Illustrations were ultimately created using the BioRender Premium^2^ platform.

## 3 Results

### 3.1 Case 1

A 39-year-old male diagnosed with acute myeloid leukemia 1 week prior was admitted to the Hematology Department on May 16, 2022. He presented with “right eye swelling, pain, blurred vision, and headache for 3 days.” Physical examination revealed right eye vision at 1 meter finger count, eyelid swelling, subcutaneous congestion, and mild conjunctival hyperemia and edema. Laboratory tests showed white blood cells (WBC) at 113.45 × 10^9^/L (3.50–9.50 × 10^9^/L), platelets at 5 × 10^9^/L (125–350 × 10^9^/L), and neutrophils at 13.94 × 10^9^/L (1.80–6.30 × 10^9^/L). Intraocular pressure was T + 1. The anti-infection regimen of “piperacillin-tazobactam + daptomycin + voriconazole + posaconazole” was prescribed, and mannitol was administered to reduce intracranial pressure. Hyperharpartonine, cytarabine, and gilteritinib were used in combination for chemotherapy.

One week later, the patient experienced worsening swelling and pain in the right eye, with ulceration and subcutaneous cyanosis noted at the inner canthus. A repeat blood test showed the WBC count of 23.15 × 10^9^/L (3.50–9.50 × 10^9^/L), C-reactive protein (CRP) at 7.95 mg/L (0.00–6.00 mg/L), and neutrophil percentage and absolute values were not measured. Given the deterioration of the patient’s condition, an infectious disease consultation was sought, and the anti-infection regimen was escalated to “vancomycin + biapenem + amphotericin B.” Supportive therapies including platelet elevation, blood product transfusion, and albumin supplementation were also provided.

**Figure 1:**
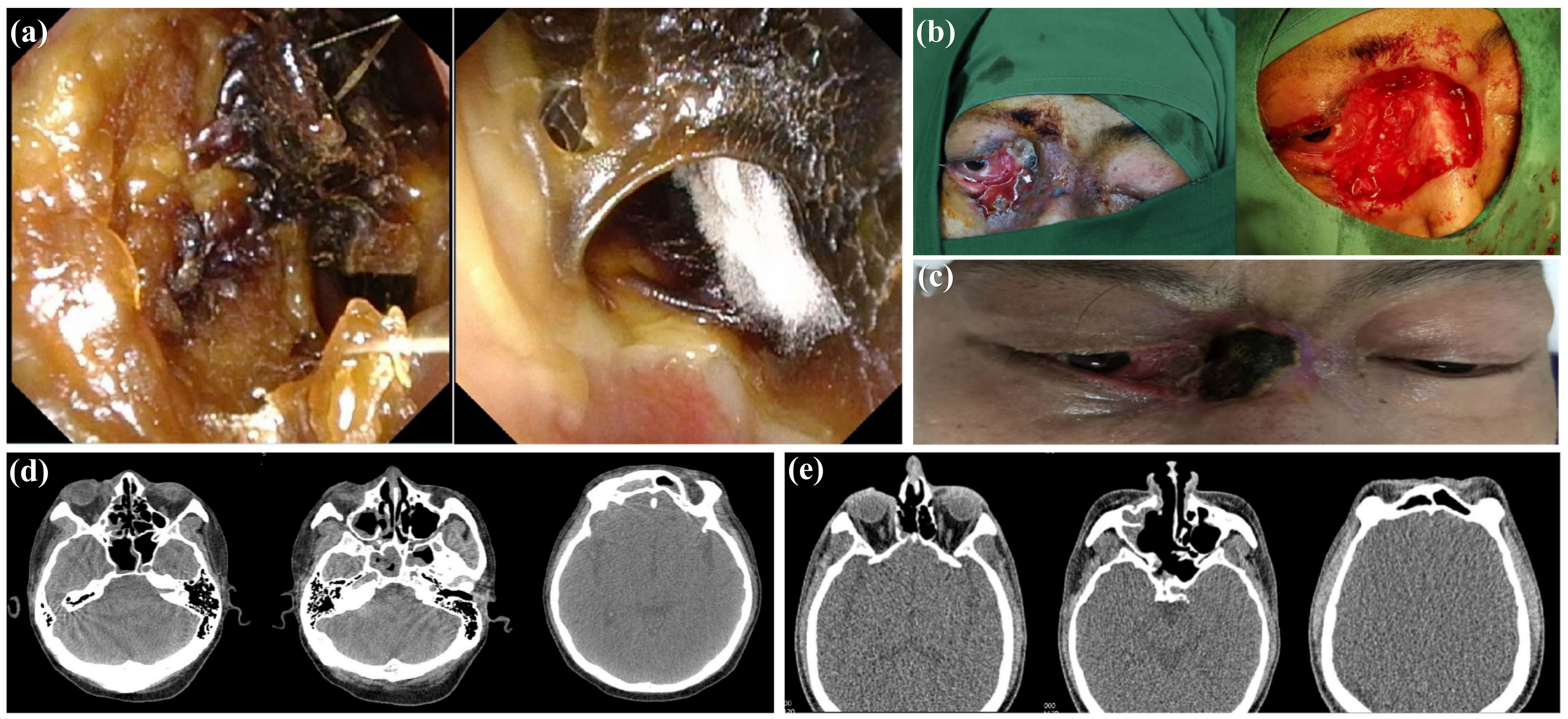
Intranasal endoscopic findings, intraoperative pathological diagnosis and preoperative and postoperative appearance of the patient. **(a)** Preoperative nasal endoscopy showed black crusts with white hyphae in the right nasal cavity, in line with typical features of mucor. **(b)** In the initial surgery, the patient’s right eyelid developed an ulcer, and the procedure involved extensive excision of the affected skin and soft tissues. **(c)** Good recovery, disappearance of swelling and formation of crusts on the surface skin at 3 months after surgery. **(d)** Preoperative sinus CT showed soft tissue necrosis in the right eye and soft tissue density shadows in the right maxillary, sphenoid, and frontal sinuses. **(e)** Postoperative sinus CT showed that necrotic tissue in the right eye was thoroughly cleaned and postoperative changes in the right maxillary, sphenoid, and frontal sinuses were observed.

Two weeks later, the patient developed undulating fever, fever peak temperature 40 °C, right periorbital ecchymosis, further aggravation of skin ulceration, and swelling of the left eye. Blood tests showed the WBC at 0.34 × 10^9^/L (3.50–9.50 × 10^9^/L), CRP at 43.9 mg/L (0.00–6.00 mg/L), and neutrophil percentage and absolute values of zero. No abnormalities were found in cell culture, blood culture, bacterial culture, or drug sensitivity testing. An otolaryngology consultation was requested, and nasal endoscopy revealed a large scab in the right nasal cavity and a white fungus-like object in the middle nasal meatus (Figure 1a). Considering the high likelihood of acute invasive fungal sinusitis, the patient underwent debridement of the nasal and orbital regions, combined eyelid fissure repair, comprehensive endoscopic sinus surgery involving ethmoidectomy (anterior and posterior), maxillary antrostomy, sphenoidotomy, and frontal sinusotomy on the right side, combined with debridement of the orbital regions (Figure 1b). Postoperative pathology confirmed acute and chronic mucosal inflammation with extensive hemorrhage and necrosis, and visible fungal hyphae and spores on the superficial necrotic surface. Fungal culture identified Mucor. Postoperatively, amphotericin B was used to irrigate the surgical cavity, and oral isavuconazole was administered at a dosage of 200 mg every 8 h for the first 2 days, followed by 200 mg once daily from the third day onward.

One month later, the swelling of the right eye significantly reduced, and immune function improved, but purulent secretions persisted in the inner canthus of the right eye. A second operation was performed: endoscopic resection of lesions in the right sinus, partial resection of the right orbital wall, and opening of the left sphenoid sinus. Postoperatively, the patient’s symptoms markedly improved, and they were subsequently discharged. Three months later, skin defects and scabs were observed at the back of the nose and the inner canthus of the right eye (Figure 1c). Sinus CT confirmed preoperative and postoperative changes in the right nasal cavity and sinus (Figures 1d,e). Currently, the patient’s symptoms have completely resolved, but acute myeloid leukemia has recurred, and he was receiving treatment.

### 3.2 Case 2

A 40-year-old female patient presented to our hospital as an emergency with a 10-day history of pain in her right upper first incisor tooth and a 5-day history of right infraorbital swelling. On admission, physical examination revealed blurred vision in the right eye, marked infraorbital swelling with high skin tension, erythema, and warmth, accompanied by a palpable sense of fluctuation. The gingival canal at the root apex of the upper right incisor exhibited a pale yellow, putrid odor accompanied by an overflow of pus. The right vestibular groove of the maxilla was significantly swollen and tender. Laboratory findings included elevated WBC count of 10.9 × 10^9^/L (3.50–9.50 × 10^9^/L), platelet count of 607 × 10^9^/L (125–350 × 10^9^/L), neutrophil count of 7.75 × 10^9^/L (1.80–6.30 × 10^9^/L), and CRP at 13.33 mg/L (0.00–6.00 mg/L).

**Figure 2:**
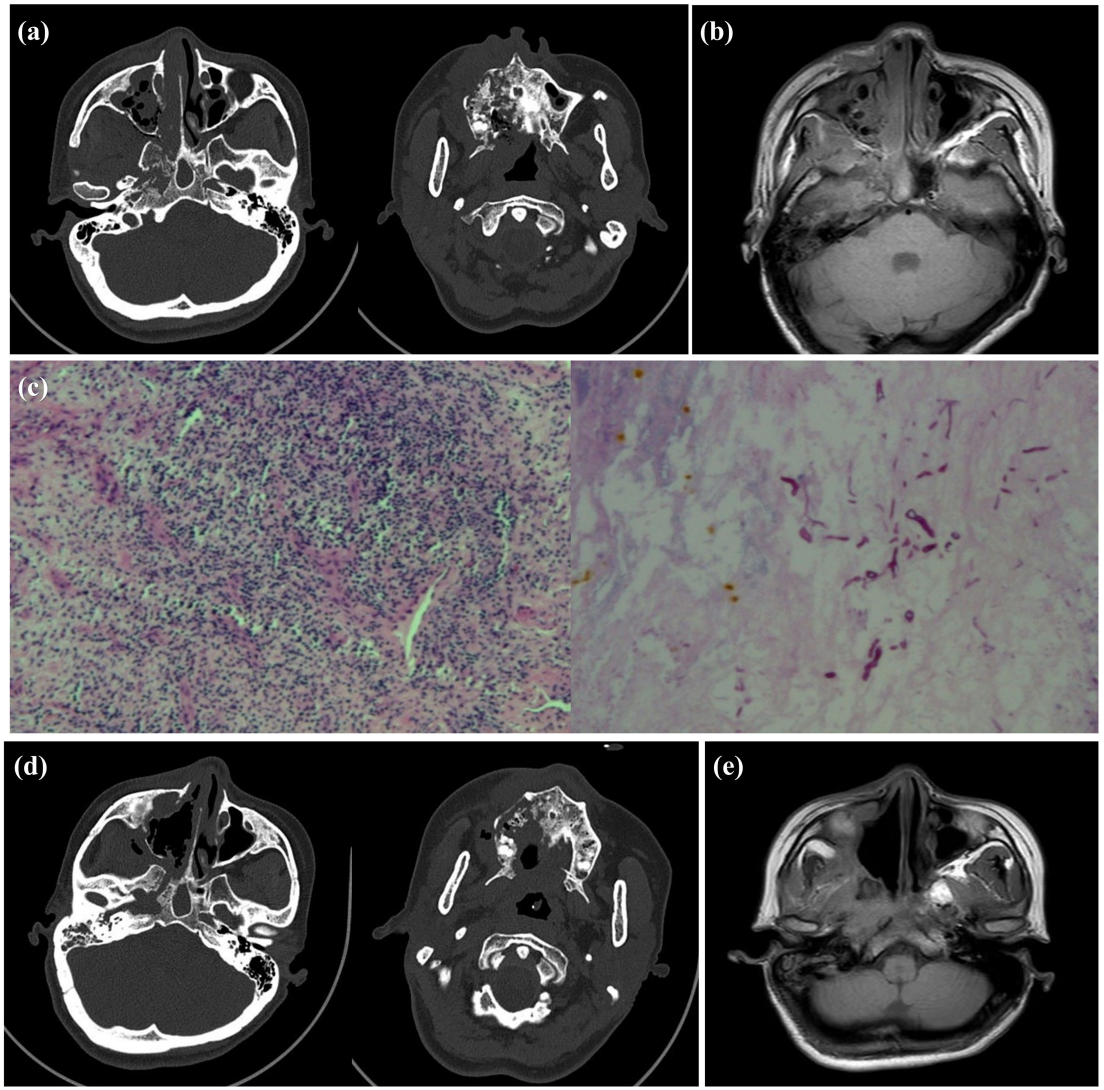
Preoperative and postoperative imaging examination and intraoperative pathology of the patient. **(a)** Preoperative sinus CT showed worm-like bone destruction on the right side. **(b)** Preoperative MRI showed a large amount of necrotic tissue in the right nasal cavity. **(c)** Intraoperative pathology confirmed the presence of Mucor hyphae, preoperative MRI revealed extensive necrotic tissue in the right nasal cavity. **(d)** Postoperative CT review indicated that dead bone was almost completely removed. **(e)** Two months after the second surgery, MRI showed recovery was good, with inflammatory soft tissue nearly resolved.

Sinus CT results indicated: (1) Soft tissue density shadows in the right nasal cavity and maxillofacial region with multiple areas of bone destruction, suggestive of possible malignancy. (2) Inflammation was present in bilateral maxillary, ethmoid, sphenoid, and right frontal sinuses. (3) Right middle ear mastoiditis (Figure 2a). MRI results confirmed: Abnormal signal shadows in the right nasal cavity and maxillofacial region with surrounding bone destruction, highly suspicious for malignant tumor. Thickening of the right nasopharyngeal lateral wall and abnormal signals in the right temporal lobe suggested tumor invasion (Figure 2b). Based on these findings, the preliminary diagnosis included: (1) Possible malignant tumor of the nasal cavity and sinuses. (2) Suborbital space infection. (3) Type 2 diabetes mellitus.

Following preoperative evaluation, the patient underwent endoscopic nasal surgery and intraoperative biopsy. Histopathological examination of frozen sections revealed extensive inflammatory necrosis and suppurative exudate, with visible mucor clusters. Special staining showed PAS (+) (Figure 2c). Given the absence of skull base invasion, extensive debridement was performed. Due to the risks associated with complete debridement in a single operation, two staged procedures were planned. For the first operation, we adopted the right nasal cavity and paranasal sinus lesion resection under nasal endoscopy, total paranasal sinus opening, orbital abscess incision and drainage, and inferior turbinate resection. Postoperative treatment with “linezolid + isavuconazole” was administered. Specifically, isavuconazole was given orally at a dose of 200 mg every 8 h for 2 days, followed by 200 mg daily for 1 month. The patient’s general condition improved significantly, with resolution of headache and nasal pain, and improvement in right-sided vision. On physical examination, the nasal cavity and oral cavity were found to be connected, the necrotic pseudomembrane of the hard palate had detached, there was no periorbital bruising, and the nasal mucosa exhibited mild hyperemia and slight swelling. The patient was subsequently discharged from the hospital. Following discharge, the patient underwent regular follow-up examinations. One month later, the condition remained relatively stable, and the patient was readmitted for secondary surgical treatment. Sinus CT revealed: Absence of soft tissue density in the right nasal cavity compared to the previous scan, unchanged soft tissue density in the right maxillofacial region, and progression of surrounding bone destruction (Figure 2d). Then, under general anesthesia, navigation-assisted endoscopic radical resection of the right maxillary sinus and debridement of necrotic tissue in the cheek, pterygopalatine fossa, and infratemporal fossa were performed. Twelve days postoperatively, the patient’s vision had recovered, but right facial paralysis persisted without other discomforts. Oral isavuconazole treatment was continued for 1 month post-discharge. Apart from right facial paralysis, the patient reported no other symptoms. Follow-up sinus MRI revealed: (1) Multiple bone destruction remained unchanged. Sinusitis showed local improvement. (2) Changes in the right maxillofacial and parapharyngeal spaces, consistent with infectious lesions, with disappearance of the original abnormal signal in the right maxillofacial space (Figure 2e). Physical examination confirmed that the palatal fistula and cheek swelling had resolved, while facial paralysis persisted.

### 3.3 Literature review

**Figure 3:**
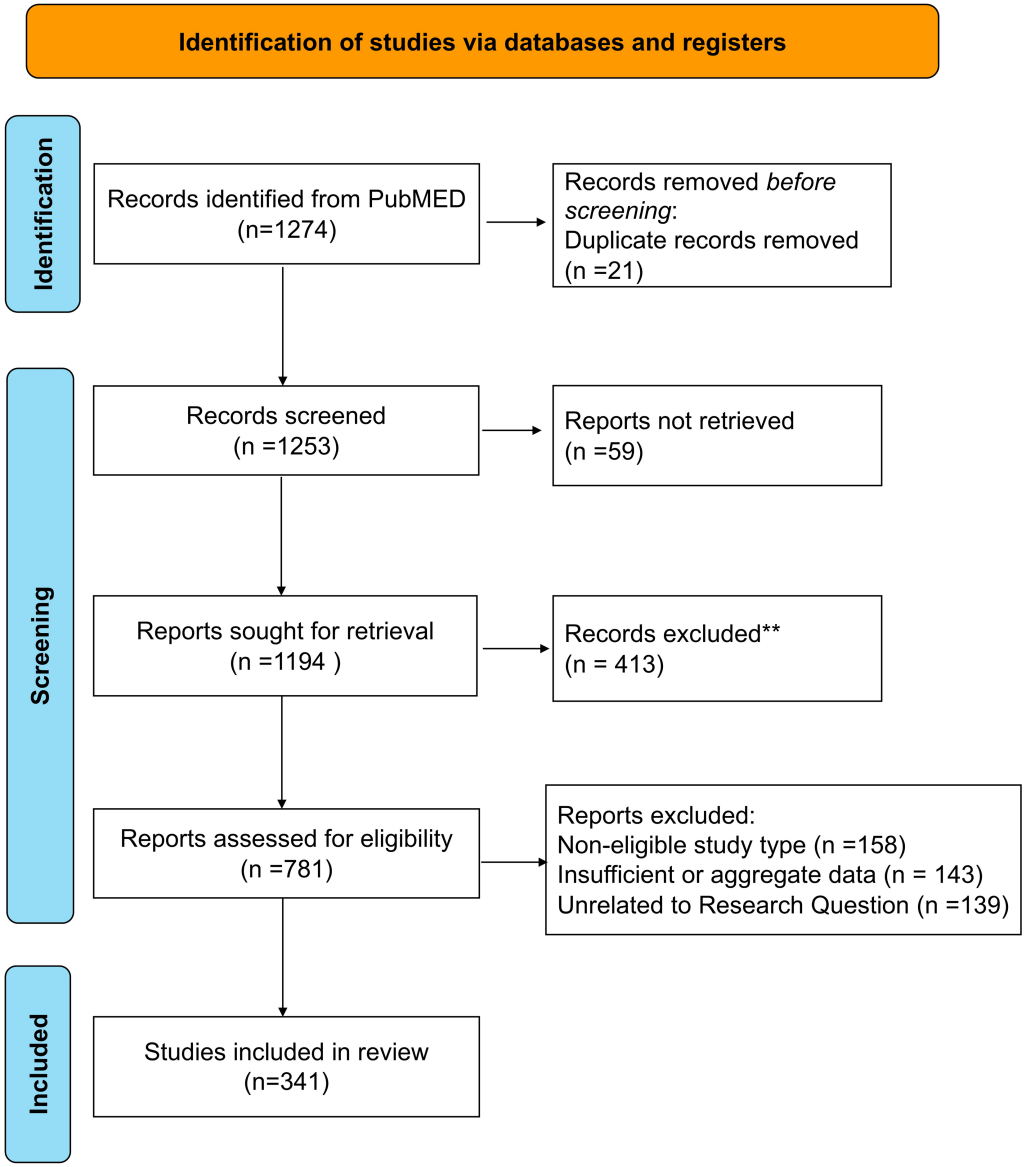
Flow chart describing the study selection process.

Initially, 1,274 records were retrieved from the PubMed database. Twenty-one duplicate records were excluded to ensure that subsequent screening was based on unique literature. Of the remaining 1,253 records, 59 reports could not be obtained due to issues with literature sources, leaving 1,194 reports for further acquisition. Four hundred and thirteen of these were subsequently excluded due to low relevance following an initial assessment. The eligibility of the remaining 781 reports was then evaluated using strict criteria. Three hundred and ninety-four articles were excluded for reasons including inconsistent research type (158 articles), data issues (143 articles), and irrelevance to the research question (139 articles). This rigorous evaluation ensured that only documents meeting the analysis requirements were included. Ultimately, 341 studies were selected for inclusion in the review and will undergo comprehensive evaluation (Figure 3). The main clinical data gathered from the selected studies are summarized (Supplementary Tables S1, S2).

A total of 387 cases were included in 341 documents and they were included in the analyzed studies. Among these, 136 cases were diagnosed with Mucor infection and 136 with Aspergillus infection. Of the 387 patients, 249 were male (64.3%) and 138 were female (35.7%). Among these patients, 13 cases (5.2%) did not provide detailed age information. A total of 155 cases (40%) presented with impaired immune function, including hematological disorders such as acute and chronic leukemia, autoimmune diseases such as systemic lupus erythematosus and rheumatoid arthritis, acquired immune deficiency syndrome, and post-organ transplantation conditions. Fungal infections combined with diabetes accounted for 14% of all cases. Among patients with Mucor infection, the diabetes comorbidity rate was 21.6%, aligning closely with previously published findings. In contrast, among those with Aspergillus infection, the rate was only 10%, significantly lower. This distinction underscores the differential distribution of risk factors across fungal etiologies.

**Figure 4:**
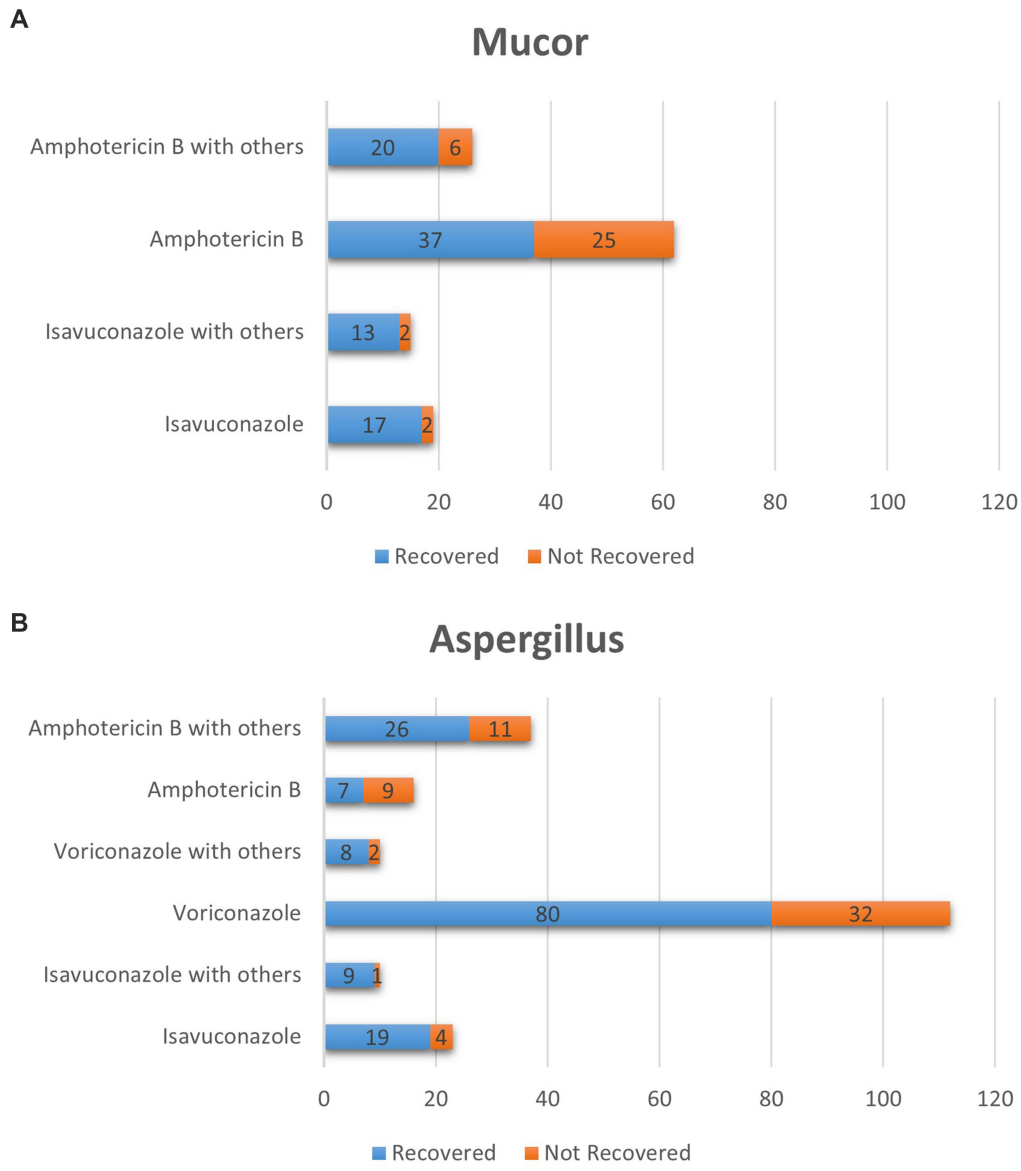
The number of cured cases of Mucor and Aspergillus genera by different drugs. **(a)** Subgroup analysis was conducted on the collected cases of Mucor genera infection, which could be divided into four subgroups. The amphotericin b combination medication group, Amphotericin b monotherapy group, the isavuconazole combination medication group, isavuconazole monotherapy group. **(b)** Subgroup analysis was conducted on the collected cases of Aspergillus genera infection, which could be divided into six subgroups. The amphotericin b combination medication group, Amphotericin b monotherapy group, the voriconazole combination medication group, voriconazole monotherapy group, the isavuconazole combination medication group, isavuconazole monotherapy group.

The patient cohort was systematically categorized into two main groups: those with Mucor infection and those with Aspergillus infection (Figure 4). Within the Mucor infection group, four subgroups were further identified. The average age of patients infected with Mucor was 41 ± 31.11 years, while that of patients infected with Aspergillus was 32.5 ± 29.99 years. Since the sample size in some subgroups was less than 5 cases, we chose Fisher’s exact test to calculate whether there were significant differences, the efficacy rate of isavuconazole monotherapy (89.47%) was significantly higher than that of amphotericin B monotherapy (59.68%), *p* < 0.05. Similarly, the efficacy rate of isavuconazole combination therapy (86.67%) was significantly superior to that of amphotericin B monotherapy (59.68%), *p* < 0.05. Amphotericin B combination therapy demonstrated a marginally better efficacy rate (76.92%) compared to amphotericin B monotherapy (59.68%), *p* < 0.05.

**Figure 5:**
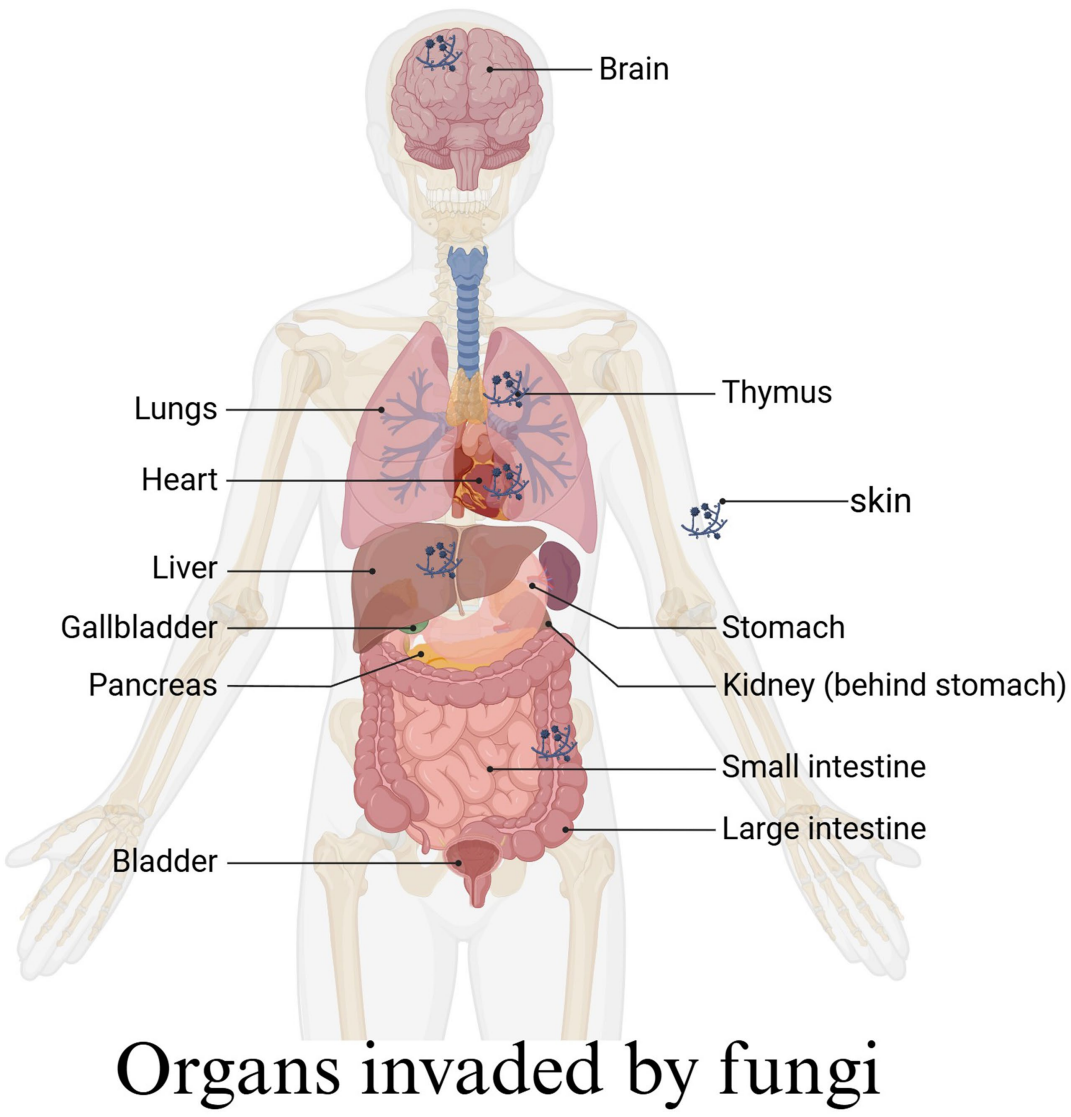
Fungi invade different organs of the human body.

Analysis of lesion involvement indicated that fungal infections can affect various organs and tissues throughout the body, with the respiratory system being the most commonly affected. Specifically, the lungs and bronchi were identified as high-risk infection sites, followed by the nasal cavity. In more severe cases, the infection may spread from the nasal cavity to the brain. Other less frequently affected sites included the gastrointestinal tract within the digestive system, the kidneys and bladder in the urinary system, as well as the skin, muscles, and bones (Figure 5).

**Figure 6:**
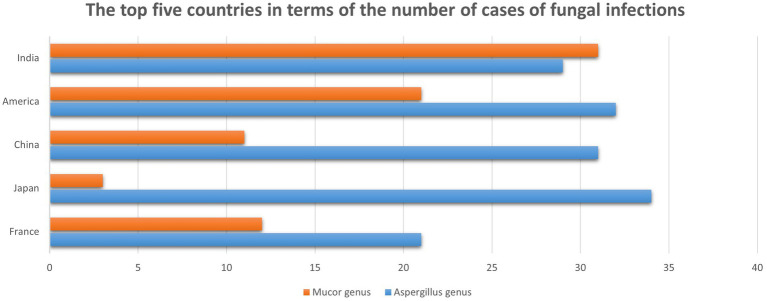
The top 5 countries with the most cases of Mucor and Aspergillus genera infections among the collected cases.

The prevalence of fungal infections caused by Mucor and Aspergillus exhibits regional variation across the globe, with higher incidence rates observed in low- and middle-income countries. Among the reported cases, the number of mucormycosis cases, in descending order, was highest in India, followed by the United States, France, China, and Japan. For aspergillosis, the number of cases, from highest to lowest, was reported in Japan, the United States, China, India, and France (Figure 6).

## 4 Discussion

The case presented herein exemplifies the potential for acute and invasive fungal infections. Our patients, adults with compromised immune systems, represent one of the most common triggers of AIFRS infection. This highlights the capacity of AIFRS to initiate sudden attacks and develop life-threatening complications. In both cases, the condition progressed rapidly from the nasal cavity to the periorbital structures, leading to the accumulation of surrounding soft tissues and functional impairment of adjacent organs. Furthermore, this report underscores the fungi associated with AIFRS infection: Mucor and Aspergillus. For different fungal species, targeted antifungal drugs should be selected; however, broad-spectrum antifungal agents remain the preferred choice for empirical therapy prior to the completion of fungal culture.

This report further emphasizes the importance of preventive measures for individuals with impaired immune function against fungal infections. According to the reviewed literature, the prevalence of immune dysfunction among affected individuals is as high as 40%. Notably, chemotherapy patients with hematological malignancies and those on long-term immunosuppressants post-organ transplantation constitute a significant proportion.

Another major risk factor is diabetes. Although the overall diabetes prevalence in our cohort is somewhat lower than some historical reports, this observation reflects the heterogeneous nature of patients with acute invasive fungal sinusitis and the multifactorial profile of associated risk factors. Diabetes remains a significant predisposing condition for Mucor infection, especially in Asian populations. Elevated blood glucose levels provide a rich carbon source for fungi such as Mucor, accelerating their proliferation. Additionally, chronic hyperglycemia induces vascular endothelial damage and microcirculatory disorders, resulting in local tissue ischemia and hypoxia, thereby reducing mucosal barrier function and facilitating fungal invasion into blood vessels and surrounding tissues (17). However, in patients with Aspergillus infection or profound immunosuppression, other risk factors—such as agranulocytosis and post-transplant immunosuppression—may play a more dominant role. Future studies should employ stratified analyses by fungal species and geographic region to further elucidate the epidemiology of risk factors in this patient population.

Although the overall diabetes prevalence in our cohort is somewhat lower than some historical reports, this observation reflects the heterogeneous nature of patients with acute invasive fungal sinusitis and the multifactorial profile of associated risk factors. Diabetes remains a significant predisposing condition for Mucor infection, especially in Asian populations. However, in patients with Aspergillus infection or profound immunosuppression, other risk factors—such as agranulocytosis and post-transplant immunosuppression—may play a more dominant role. Future studies should employ stratified analyses by fungal species and geographic region to further elucidate the epidemiology of risk factors in this patient population.

Environmental factors play a critical role in fungal infections (18). Indoor infections typically occur in areas with high humidity and poor ventilation, while outdoor infections are more prevalent in warm, dusty environments near water bodies (19). Among the reviewed cases, individuals with compromised immune function exposed to these conditions exhibit an increased risk of fungal infections due to skin contact or superficial tissue damage.

Acute and chronic invasive fungal infections caused by Mucor and Aspergillus can affect various organs (20). In our reviewed cases, these primarily include the five senses, skin, lungs, gastrointestinal tract, bladder, spleen, etc. AIFRS represents the most severe manifestation of sinusitis caused by fungal infections, often requiring extensive surgical resection combined with antifungal therapy (21). Due to the complex anatomical structure of the facial cavities and their proximity to vital organs, blood vessels, and nerves, achieving a cure through surgery alone incurs substantial costs for patients. Antifungal drugs enable the restriction of fungal infections within the body and facilitate lesion reduction until clearance (12, 22).

The selection of antifungal drugs is a pivotal aspect of treatment. The use of antifungal agents dates back to the 1950s, with polyenes such as Amphotericin B being the cornerstone of therapy for invasive systemic fungal infections (23, 24). Despite its efficacy, Amphotericin B is associated with significant side effects, prompting the development of newer classes of antifungal drugs, including azoles and echinocandins (25). Each new generation of drugs addresses deficiencies in the previous generation’s resistance to certain fungi (26). Distinguishing fungal metabolic and signaling pathways from those of mammalian cells remains a key focus for developing more effective and less toxic antifungal agents (27). For instance, first-generation triazole drugs, fluconazole and itraconazole, demonstrate better activity profiles against specific fungi. However, drug resistance, dangerous drug interactions, and toxicity limit their utility in treating fungal infections (26).

To overcome these limitations, second-generation triazole drugs were developed, including voriconazole, posaconazole, and isavuconazole (28). Voriconazole, a derivative of fluconazole, can be administered orally and intravenously with similar pharmacokinetic characteristics. Posaconazole, derived from itraconazole, exhibits broader efficacy and is used to treat Aspergillus-, Fusarium-, and Mucor-related infections (29). Isavuconazole, the active metabolite of the prodrug isavuconazole ammonium sulfate, appears to have an activity spectrum comparable to voriconazole and posaconazole but demonstrates fewer drug interactions and lower nephrotoxicity, hepatotoxicity, visual effects, and neurotoxicity (30). It can serve as a primary prophylactic agent (31). Prospective studies confirm its efficacy and safety in primary prevention (32). For example, Bose et al.’s single-center, open-label, prospective Phase 2 study demonstrated that among 65 patients receiving isavuconazole prophylaxis (95% with AML), the incidence of possible and probable invasive fungal infections was only 15% (33). Another prospective study revealed that among 95 patients treated with isavuconazole, only 3 cases (3.1%) experienced invasive fungal infections (34). Thus, isavuconazole appears to be the best-tolerated among the three second-generation azole drugs currently available. Although its clinical experience is relatively limited compared to voriconazole or posaconazole, and several toxicities may require years of clinical observation to manifest, isavuconazole exhibits the least complex drug interaction profile among the three (35).

## 5 Conclusion

In the described cases, both patients received isavuconazole pre- and post-surgery, achieving favorable prognostic outcomes. Literature comparisons further validate these findings, demonstrating that the efficacy of the new triazole drug isavuconazole in treating invasive aspergillosis and mucormycosis significantly surpasses that of amphotericin B, with efficacy comparable to voriconazole (*p* < 0.05). These results provide valuable guidance for clinical medication decisions.

## Author’s note

Yuwan Song contributed to this work during his master’s studies at Yantai Yuhuangding Hospital and continued with revisions during his doctoral studies at Charité – Universitätsmedizin Berlin. Both affiliations are listed to reflect this.

## Data Availability

The original contributions presented in the study are included in the article/Supplementary material, further inquiries can be directed to the corresponding authors.

## Footnotes

^1^ https://pubmed.ncbi.nlm.nih.gov/

^2^ https://app.biorender.com/
